# SMaSH: a scalable, general marker gene identification framework for single-cell RNA-sequencing

**DOI:** 10.1186/s12859-022-04860-2

**Published:** 2022-08-08

**Authors:** M. E. Nelson, S. G. Riva, A. Cvejic

**Affiliations:** 1 European Bioinformatics Institute, Wellcome Genome Campus, Cambridge, CB10 1SD UK; 2 Department of Haematology, University of Cambridge, Cambridge, CB2 0AW UK; 3 Wellcome - Medical Research Council Cambridge Stem Cell Institute, Cambridge, CB2 0AW UK; 4 Open Targets, Wellcome Genome Campus, Cambridge, CB10 1SA UK; 5 Wellcome Sanger Institute, Wellcome Genome Campus, Cambridge, CB10 1RQ UK

**Keywords:** Single-cell RNA-sequencing, Marker genes, Feature selection

## Abstract

**Background:**

Single-cell RNA-sequencing is revolutionising the study of cellular and tissue-wide heterogeneity in a large number of biological scenarios, from highly tissue-specific studies of disease to human-wide cell atlases. A central task in single-cell RNA-sequencing analysis design is the calculation of cell type-specific genes in order to study the differential impact of different replicates (e.g. tumour vs. non-tumour environment) on the regulation of those genes and their associated networks. The crucial task is the efficient and reliable calculation of such cell type-specific ‘marker’ genes. These optimise the ability of the experiment to isolate highly-specific cell phenotypes of interest to the analyser. However, while methods exist that can calculate marker genes from single-cell RNA-sequencing, no such method places emphasise on specific cell phenotypes for downstream study in e.g. differential gene expression or other experimental protocols (spatial transcriptomics protocols for example). Here we present SMaSH, a general computational framework for extracting key marker genes from single-cell RNA-sequencing data which reliably characterise highly-specific and niche populations of cells in numerous different biological data-sets.

**Results:**

SMaSH extracts robust and biologically well-motivated marker genes, which characterise a given single-cell RNA-sequencing data-set better than existing computational approaches for general marker gene calculation. We demonstrate the utility of SMaSH through its substantial performance improvement over several existing methods in the field. Furthermore, we evaluate the SMaSH markers on spatial transcriptomics data, demonstrating they identify highly localised compartments of the mouse cortex.

**Conclusion:**

SMaSH is a new methodology for calculating robust markers genes from large single-cell RNA-sequencing data-sets, and has implications for e.g. effective gene identification for probe design in downstream analyses spatial transcriptomics experiments. SMaSH has been fully-integrated with the ScanPy framework and provides a valuable bioinformatics tool for cell type characterisation and validation in every-growing data-sets spanning over 50 different cell types across hundreds of thousands of cells.

**Supplementary Information:**

The online version contains supplementary material available at 10.1186/s12859-022-04860-2.

## Background

**Fig. 1:**
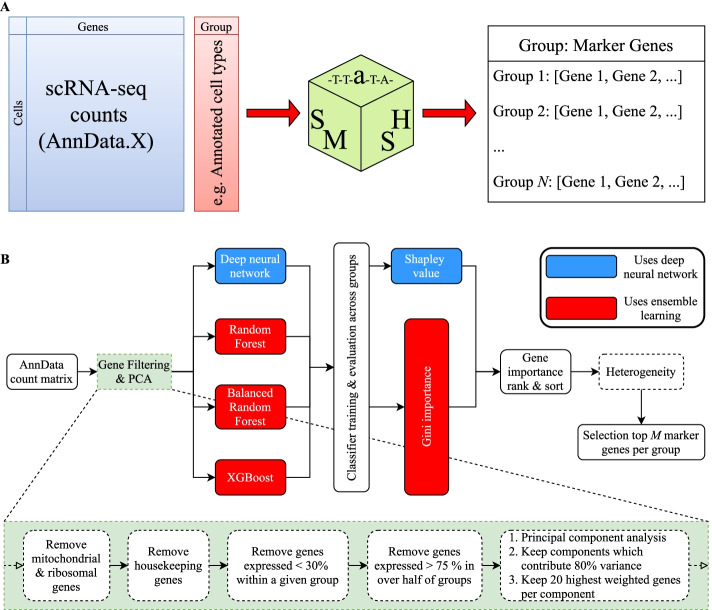
The SMaSH framework. **A** SMaSH works directly from the counts matrix, producing a dictionary relating the user-defined classes of interest (e.g. cell type annotations) to top marker genes for each class (default top 5). **B** SMaSH filters and ranks genes according to an ensemble learning model or a deep neural network

Single-cell RNA-sequencing (scRNA-seq) [1, 2] is advancing our understanding of gene expression at the single-cell level in a variety of biological contexts, and is particularly relevant for learning the genetic profile of known and new cell types in a variety of different biological contexts at unprecedented resolution. In scRNA-seq of human tissue, it is usual for each cell to be aligned against a reference genome comprising over 20,000 genes. Various downstream dimensionality reduction and manifold embedding techniques are employed to reduce the complexity of this space and identify biologically distinct clusters of single cells corresponding to known and new phenotypes. Reversing this problem, if known cell types can be identified, it is relevant to ask which genes contribute most significantly to that particular phenotype. This question of identifying a *small* and *unique* set cell type-specific marker genes for several different clusters of cells is relevant for several reasons. In one case, knowing the most important marker genes influences the parameter space of genes to compare in that cell type across a variety of different biological scenarios. An example would be the differential behaviour of marker gene for a cell type in the case of cancerious tissue versus healthy tissue. As a second, and more topical example, scRNA-seq provides no information on the location of different cell populations in tissue. Spatial transcriptomics (STx) addresses this issue by resolving the locations of the whole or part of the sequenced transcriptome, thus providing better context for studying the vast heterogeneity of different cellular states throughout different organs and tissues. Protocols such as seqFISH (sequential Fluorescence In Situ Hybridization) [3], ISS (In Situ Sequencing) [4], and MERFISH (Multiplexed Error-Robust FISH) [5] all aim to achieve this single-cell (and even sub-cellular) spatial resolution but for a limited number (i.e. a few hundred from a candidate list of typically 20,000 expressed genes) of pre-selected genes. The great utility of spatial technologies therefore entirely relies on the analysers’ abilities in selecting an optimal shortlist of candidate genes for spatial study which address their specific biological questions. These two examples illustrate the great importance of calculating marker genes for specific cell types which can uniquely identify those cells against all others in the data-set, in a statistically robust and reliable manner. It is also important that the analyser can calculate marker genes relevant for many *different* biological questions but applied to the same formal scRNA-seq data. Does the analyser want to select a sample of genes which distinguish different T cell populations from myeloid cell populations in order to study the immune response surrounding cancer? Does the analyser instead care about the behaviour of cells of different potencies (stem cell vs. progenitor vs. differentiated cell) in a small foetal tissue sample? Does the analyser care about genes which are best at distinguishing annotated tumour tissue from surrounding background tissue, a healthy control sample, and so on? Whilst computational techniques which attempt to select highly representative genes from large single-cell RNA-sequencing data-sets do exist, we are aware of no such techniques which select representative genes in a general enough manner to deal with *any* question the analyser might pose. The success of future single-cell studies which attempt to understand the biology of new and niche populations of cell types hidden in highly complex scRNA-seq data depends on the ability to highly specific genes that can label distinct cell populations in tissue sections according to any generic problem the prospective user is interested in. Moreover this must be achieved in a computationally efficient manner which is experimentally reproducible within and without the analysis framework. To this end we propose SMaSH (Scalable Marker (gene) Signal Hunter, Fig. 1) for the identification of important genes, from already annotated scRNA-seq data. This finds application in numerous single-cell downstream analyses, such as the classification of new cell types, the differential expression of those marker genes, and even the design of gene-specific probes for the emerging technologies in spatial transcriptomics.

The strength of SMaSH is that it has been designed to work with any scRNA-seq barcode annotation provided by the user, and is therefore completely generic and scalable to a whole variety of gene identification tasks. Existing techniques rely on many different metrics for measuring the relevant important of different genes. In the SMaSH protocol, we recast marker gene selection as a feature importance calculation from a central SMaSH model which uses supervised learning to classify cells in scRNA-seq data according to user-defined annotations for each cell. We demonstrate that optimal performance of SMaSH is obtained from two different modes: the *ensemble learner* mode, which uses Gini importance to rank gene significance per cell type; and the *network* mode which uses Shapley values to rank the genes.

The utility of SMaSH is evaluated against other approaches for marker genes selection from scRNA-seq data(e.g. see  [6, 7]). These approaches identify marker genes based only on their expression profiles throughout the tissue of interest. This can lead to identification of marker genes with wide expressions across multiple cell types and which therefore have a limited power to distinguish individual cell types within the data. The motivation for SMaSH was to select relevant, highly specific genes for bespoke downstream applications like STx, using universal non-linear functions [8], as would be obtained from algorithms with a high number of degrees of freedom (neural networks or ensemble learners). These algorithms can learn a feature space parameterised by cell type-specific genes using user-provided cell type annotations for each data point. The need for a non-linear approach stems from the inherent non-linearity in scRNA-seq gene expression profiles. This should be compared with some other gene selection methods which rely on linear algorithms, such as linear correlation functions, to select key genes. Such linear approaches risk missing valuable information in the task of selecting relevant features (genes) from a higher-dimensional feature space which is inherently non-linear. In these studies we also wanted to assess the performance of the non-linear gene selection approach with state-of-the-art linear approaches. We found that existing marker gene tools using the linear paradigm did not generalise well across different scRNA-seq data-sets when trying to determine cell type-specific genes, often generating marker genes which lead to high misclassification rates in the data to the ground-truth cell types. Often the genes were also highly non-specific to cell types even when the training set was partitioned into known cell types *a priori*. SMaSH was observed to determine marker genes which consistently well describe, classify, and distinguish the unique cell types across many data-sets, giving more support to the need for non-linear functions in gene selection in a variety of biological scenarios. This performance improvement is vital for reliable marker gene calculations, and will only become more relevant as the task of scRNA-seq turns to identifying new sub-populations of cells within known phenotypes across developmental biology, cancer, and more besides. We believe this sets the scene for establishing SMaSH as an out-of-the-box framework for bridging the gap between gene expression bioinformatics software in scRNA-seq. We also noted a lack of direct usability in some approaches with respect to popular computational pipelines, such as ScanPy [9]. Once again, SMaSH was designed to work easily with such frameworks. SMaSH can be implemented in both computer processing unit (CPU) and graphics processing unit (GPU) ‘modes’. The latter mode is relevant for analysing the ever-growing data-sets under consideration in single-cell transcriptomics commonly spanning over $10^{6}$ cells. Putting this all together, we believe SMaSH offers a valuable contribution to the field of bioinformatics software, and goes someway towards standardising the analysis workflow for scRNA-seq and other downstream protocols (e.g. STx).

Full details of the SMaSH model and procedure are provided in the “Methods” section. In particular, note that SMaSH implements its gene ranking and importance scoring using two different machine learning modes: the *ensemble* mode and the *network* mode. We originally considered three different models in the benchmarking of the *ensemble* mode: the Random Forest (RF) [10], the Balanced Random Forest (BRF) [11], and XGBoost [12]. The need for different models is a result of variations in performance across different biological data-sets and different data-modalities. However we observed consistently excellent performance from XGBoost in the majority of scenarios (see *Results*), and this therefore represents the default model for the *ensemble* mode. The *network* mode comprises a feedforward deep neural network (DNN) [13, 14]. The *ensemble* mode evaluates the importance score of each gene according to the *Gini importance* [15], whilst the *network* mode evaluates importance according to the *Shapley value* [16].

## Results

To address the performance of SMaSH, we consider the following questions:

- Can SMaSH determine highly-expressed genes which uniquely identify broad cell type populations (e.g. 10 cell types) in a variety of different scRNA-seq data-sets?
- By extension, can SMaSH identify specific marker genes in more complex data-sets comprising many cell types (above 30) in addition to the broad cell sub-types of the previous point? Such data-sets are becoming increasingly common in scRNA-seq analyses.
- Is SMaSH’s approach competitive with existing linear approaches to gene identification?
- Does SMaSH select biologically meaningful marker genes? This is assessed in two approaches: (1) cross-checking SMaSH markers against known cell type markers in literature (See Additional file 1: Tables S1, S2); (2) verifying with spatial transcriptomics that SMaSH markers can identify cell types highly-localised to specific tissue compartments (Additional file 1: Fig. S4).

To evaluate the performance of SMaSH markers, we benchmarked it against two recent standalone computational algorithms, scGeneFit [6] and RankCorr [7], which calculate marker genes from scRNA-seq data using linear programming and gene-by-gene correlations respectively. Unlike SMaSH these algorithms determine relevant markers by considering the entire scRNA-seq counts matrix, but we were still able to run them on specific cell type annotations in order to make a fair comparison with SMaSH, and the same gene sets are used in all methods. The additional ensemble learners we considered (Random Forrest and Balanced Random Forrest) when designing the *ensemble* mode also act as additional models to benchmark our default *ensemble* model (XGBoost) against.

**Table 1:** Single-cell RNA-sequencing data-sets in this study

Data-set	Technology	Cells	Genes	$\#$ Cell types	References
Human lung cancer (broad)	10X scRNA-seq	54,574	18,612	7	N.A.
Human lung cancer (cell sub-types)	10X scRNA-seq	54,574	18,612	34	N.A.
Mouse brain (broad)	Single nucleus RNA-seq	40,532	31,053	9	[17]
Mouse brain (cell sub-types)	Single nucleus RNA-seq	40,532	31,053	31	[17]
Zeisel	10X scRNA-seq	3005	4000	7	[18]
CITE-seq	CITE-seq	8617	500	13	[19]
Paul15	MARS-seq	2730	3451	10	[20]
Human foetal liver	10X scRNA-seq	65,712	19,572	18	[21]
Human foetal organs	10X scRNA-seq	211,754	23,054	40	[21]

The different data-sets considered in the benchmarking of SMaSH

We compared RankCorr, scGeneFit, and the two modes implemented within SMaSH across several publicly available data-sets: Zeisel [18], a data-set based on CITE-seq technology [19], a mouse brain single nucleus RNA-sequenced data-set [17], a healthy foetal liver data-set [21], Paul15 stem cell data [20], and a large lung cancer data-set. We also considered an extension of the foetal liver data-set covering skin and kidney cells in addition to liver when later studying the performance of SMaSH on the problem of identifying organ-specific marker genes. All data-sets are summarised in Table 1. To systematically study the effect of cell type granularity when benchmarking SMaSH, the mouse brain data-set was split into two different sets of annotations. These are ‘broad’ and a higher-granularity set where each broad cell type was further subdivided. For the healthy foetal organ data-set, which spans the kidney, liver, femur, and yolk sack, we considered both the complete scRNA-seq data spanning all of those organs and the 40 different published annotations, and also separately the liver-only cells where we applied our own set of cell annotations for that specific organ, corresponding to 18 different cell types. This was done to further study how the different marker gene frameworks responded to the same type of data but at different levels of complexity (18 distinct cell types vs. 40 in the full data-set). These different data-sets use a variety of scRNA-seq technologies and conditions and were selected to so that the methodologies described could be tested on a variety of results from different type of tissue (e.g. mouse brain vs. human lung) and different numbers of sequenced single cells (e.g. 10$^{4}$ vs. 10$^{6}$) and therefore data-set complexity. The lung cancer data-set comprised lung cancer tissue, the 5 cm of tissue surrounding the tumour, and healthy lung tissue from donors. Annotations on this final data-set were performed using a combination of principal component analysis of the highly-variable genes for dimensionality reduction and manifold learning via UMAP [22] for visualisation purposes. Confounding of the data resulting from possible batch effects was tested and removed by applying the Harmony algorithm [23] on the dimensionally-reduced cell phenotype representation. For the lung data annotations there are also two levels of annotation complexity. First we defined seven ‘broad’ cell types corresponding to myeloid cells, B cells, T cells, dendritic cells, natural killer cells, mast cells, and epithelial cells. Each of these broad cell types, with the exception of epithelial cells, was then split into additional cell sub-types, resulting in 34 distinct classes in the final analysis. Cell sub-types are determined before running SMaSH, and are motivated by prior knowledge of certain genes in specific clusters and known literature surrounding relevant genes for specific cell sub-types. Additional details on the benchmarking are described in the “Methods” section.

### SMaSH determines highly-expressed genes which uniquely identify broad cell type populations in scRNA-seq

**Fig. 2:**
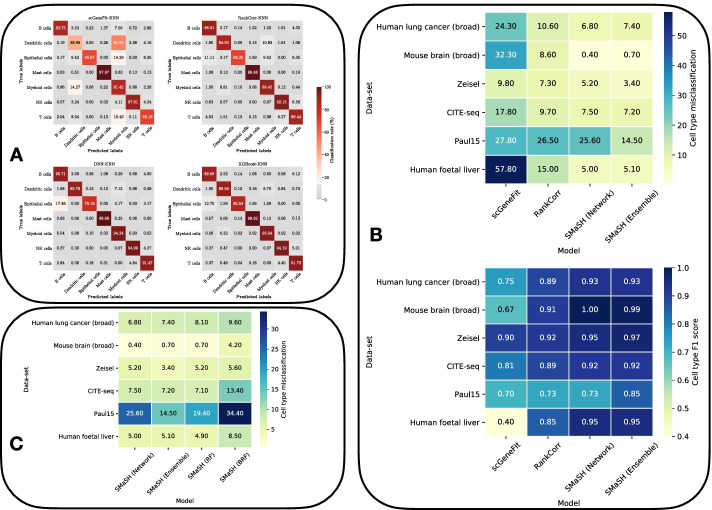
Classifying broad cell types based on SMaSH-specific marker genes. **A** Confusion matrices for the top 30 marker genes per cell type in the lung broad cell classification data-set for scGeneFit, RankCorr, SMaSH using the *network* mode, and SMaSH using the *ensemble* mode (using XGBoost). **B** Cell misclassification and $F_{1}$ scores for the two SMaSH modes against scGeneFit and RankCorr. **C** Benchmarking different SMaSH ensemble learning models across biological scRNA-seq data and related modalities

In this first set of studies, we focused on the ‘broad’ cell types covering the previously defined data-sets; cell type multiplicities vary between 7 and 18. scGeneFit, RankCorr, and SMaSH separately calculated the most important 30 marker genes (per annotation class, e.g. cell type) to classify cells according to their ground-truth annotations in each data-set and evaluated them according to our classifiers outlines in our “Methods” section. The results are summarised in Fig. 2, where we separately benchmarked the *ensemble* and *network* modes of SMaSH against existing approaches (A and B), and three different ensemble models in the *ensemble* mode of SMaSH against one-another (C).

We observe that the misclassification and general performance with SMaSH outperforms existing approaches across all data-sets. This is particularly true for larger data-sets like the lung and human foetal liver, where SMaSH offers substantially lower misclassifications across all cell types. Thus, SMaSH scales very generally to marker gene identification problems in both simple data-sets like Zeisel and in larger data-sets, which are fast becoming the norm in single-cell biology. Confusion matrices of the true-positive (classification) rates for RankCorr, scGeneFit and the *network* and *ensemble* SMaSH modes evaluated on the ground-truth 7 broad cell types in the lung data are shown in Fig. 2A). For both smaller and larger data-sets (e.g. Zeisel vs. broad lung) the two SMaSH modes perform similarly. We found that the default *ensemble* mode XGBoost model performs particularly well across all cases, and is in the top two best performing models in 5/6 data-sets, and notably in the case of the mouse brain data achieves sub-percent misclassification rates where the best recently-developed approach of RankCorr achieves an 8.6 $\%$ average misclassification (Fig. 2B). The benchmarking of this default ensemble model against the others confirms it as the strongest ensemble performer across the majority of the data-sets (Fig. 2C).

**Fig. 3:**
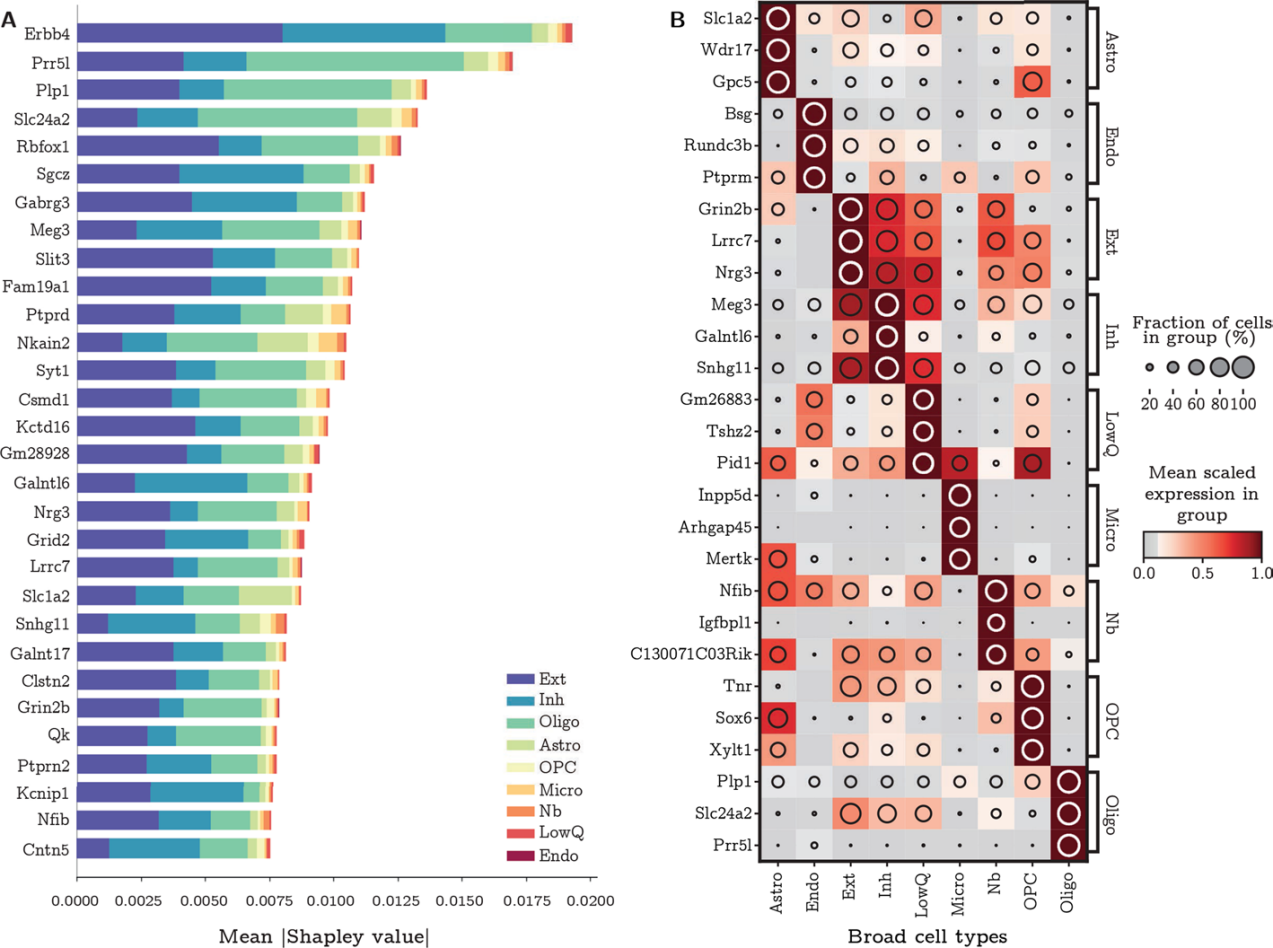
Marker genes for the broad mouse brain cell types. **A** The mean |Shapley value| for the top 30 ranked marker genes across all broad cell types of the mouse brain, before additional filtering and sorting, using SMaSH’s *network* mode. Different colours indicate the different class contributions which that particular gene explains. **B** the final three markers for each class/broad cell type are shown, with the colour profile corresponding to the mean logarithm of the gene expression and a pattern uniquely matching specific markers to specific cell types

The SMaSH implementation provides the most important marker genes for each class, based on their rank in Gini importance or Shapley value. As a concrete example, in the case of the broad mouse brain data this would correspond to unique markers per each of the 9 cell types. These cell types biologically map to astrocytes (Astro), microglia (Micro), endothelial cells (Endo), excitatory neurons (Ext), inhibitory neurons (Inh), neuroblasts (Nb), oligodendrocyte (Oligo), oligodendrocyte precursors (OPC), and a generic group of low quality cells (LowQ). These top three markers, ordered for each cell type based on their Shapley value computed by the *network* mode, are shown in Fig. 3. The companion Shapley score plot illustrates the values of high ranking genes across several different classes. In most cases, SMaSH is able to identify key genes which are uniquely (or nearly uniquely) expressed in one particular cell type of interest relative to all others. The colour scale, corresponding to the mean logarithm of gene expression, is normalised to between 0 and 1.0, where dark brown indicates very high levels of gene expression. Three dark brown populations can be uniquely generated for each cell type, indicating that highly and uniquely expressed genetic markers are present. Such markers would be useful for exclusively tagging particular cell types in the design of protocols for single-cell spatial resolution of mRNA. This gives SMaSH an advantage over existing approach which, although also able to select marker genes, the SMaSH markers are more unique to specific cell types.

### SMaSH identifies specific marker genes in more complex data-sets comprising many cell types and sub-types in lung cancer patients and mouse brain cells

**Fig. 4:**
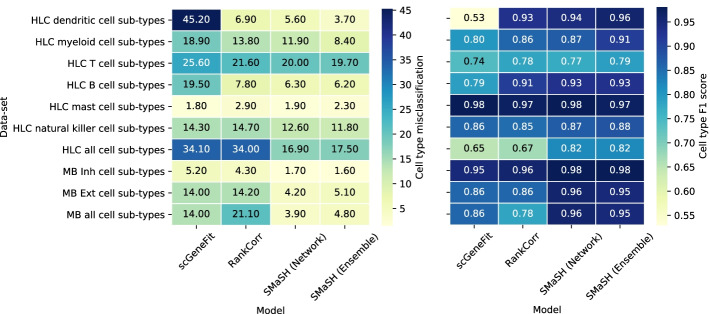
Marker gene misclassification rates and $F_{1}$ scores in cell types in the lung and mouse brain. Performance for each human lung cancer cell sub-type and framework, including the two modes in SMaSH. HLC: Human lung cancer; MB: Mouse brain

One challenge in scRNA-seq gene identification is determining genes with the greatest statistical power for distinguishing increasingly complex and granular cell-type identifications. In the lung data-set each of the broad cell types can be further subclassified into several biologically distinct cell types. We repeated the misclassification calculation for 6 of the 7 broad lung cell types which can be further sub-divided, separately determining the top 30 markers for each of these 6 classification problems from the broad cell into its sub-types. We also evaluated this as a single classification problem, directly calculating the top 30 markers for classifying the entire lung data-set cells directly into their 34 lung cell sub-types. We evaluated SMaSH against existing approaches for identifying relevant markers, finding substantial reduction in misclassification rate compared to current methods. This was observed in both the ‘two-step’ approach of first classifying into the broad cell types, and then sub-classifying them, and the ‘one-step’ approach of directly classifying cells into the distinct 34 sub-types. We found that the misclassifcation rates for the ‘one-step’ problem were generally higher than the ‘two-step’ across all models. This is not unexpected given the added complexity of performing a 34-class problem directly and indicates that better marker gene extraction can be obtained by splitting the cell classification problem into two or more sub-problems. Moreover, we found that the largest gains in the ‘two-step’ problem are provided by either a more non-linear model, the deep neural network of our *network* mode, or XGBoost as our default model in the *ensemble* mode. These comparisons are summarised in Fig. 4, where we also considered the same ‘one-step’ and ‘two-steps’ marker gene identification approach in the mouse brain data-set. Substantial performance improvements are observed with SMaSH.

**Fig. 5:**
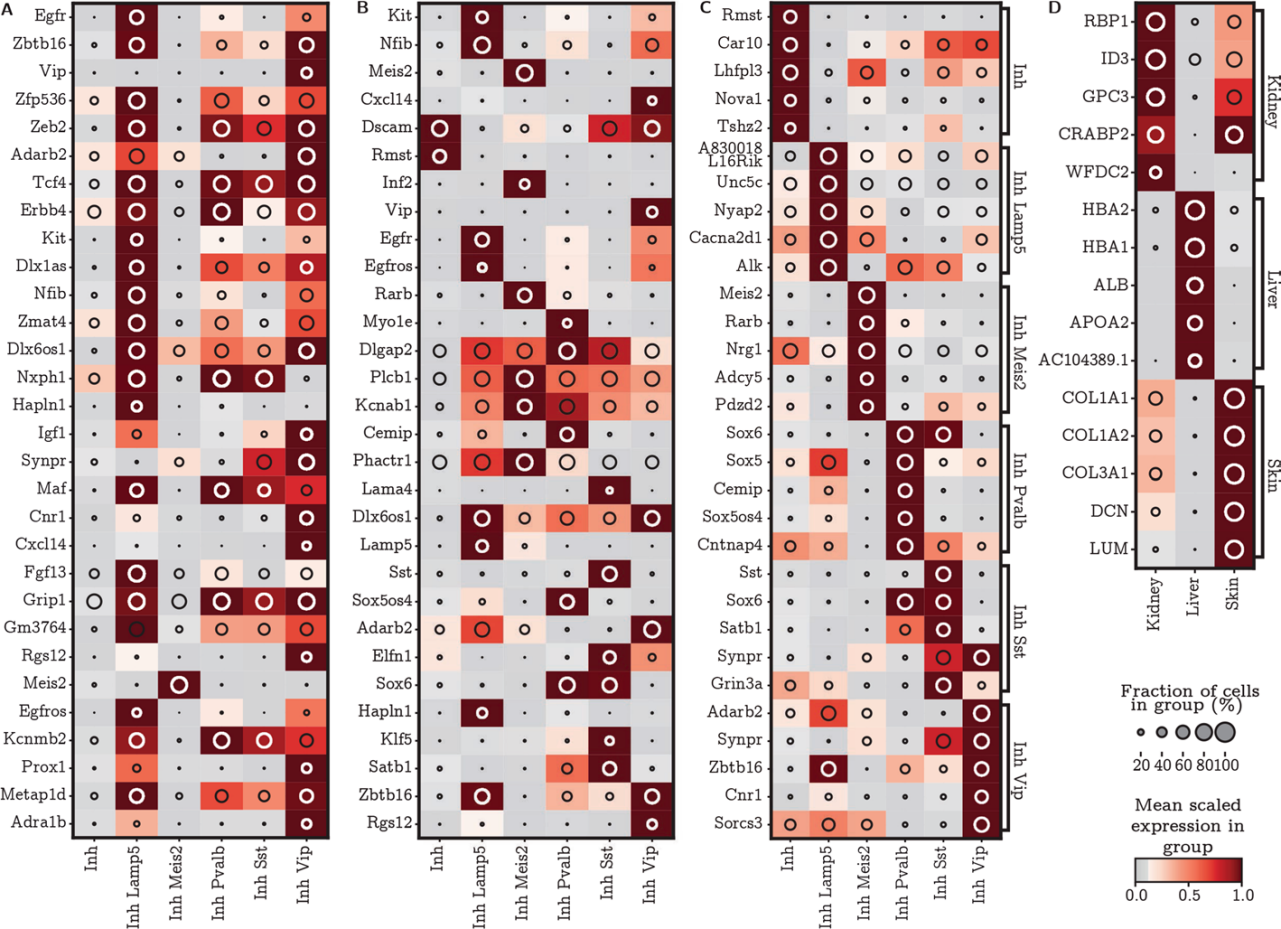
Marker genes for the mouse brain cell sub-types from the Inhibitory neuron broad types, and human foetal organ of origin classification. The mean logarithm of gene expression for mouse brain cell Inhibitory neuron cell sub-type markers. **A** the markers for scGeneFit; **B** the markers for RankCorr; **C** markers from SMaSH’s *network* mode. Particularly in the case of SMaSH unique patterns can still be identified in this highly granular cell-type identification problem, whereas approaches such as scGeneFit are not able to identify many markers which uniquely resolve the sub-types present. **D** SMaSH is able to select statistically significant markers for a highly imbalanced problem of distinguishing organs of origin in foetal scRNA-seq

We also observe that SMaSH is still able to identify important marker genes which distinguish individual cell sub-types even when they belong to the same broad classification, as demonstrated for e.g. the sub-types of the mouse brain Inhibitory neuron broad types in Fig. 5. For this Figure, the markers are calculated using the *network* mode of SMaSH (C), which identifies more unique markers compared with the benchmarked approaches.

### SMaSH generalises to non-cell-type-specific marker gene problems

In this section we demonstrate how SMaSH can be readily applied to very general marker gene identification problems in scRNA-seq. Thus far SMaSH has been implemented in problems for selecting marker genes to distinguish different cell types, which has obvious utility in spatial transcriptomics. However, this same procedure can be repeated in very general annotations and we illustrate this by taking a stratified sample of a publicly available foetal organ data-set [21] and calculating marker genes which best distinguish three different organs of origin. These are kidney, liver, and skin, using those organs now as the relevant annotations for each cell. Inspite of the naturally imbalanced nature of such data, SMaSH is still able to identify statistically significant markers for specific organs. The markers identified uniquely (or nearly uniquely) describe the particular organ of interest versus the other two in the classification problem (Fig. 5D). The misclassification rates are $F_{1}$ scores are summarised across all four models considered in SMaSH (three different ensemble learners in the *ensemble* mode) in Table 2, and the top two performing models are found to be the defaults for the SMaSH *network* and *ensemble* modes. The markers extracted from the top-scoring models were also confirmed to be highly relevant to the particular organ of interest following a cross-check of their function in relevant biological literature (see Additional file 1: Tables S1, S2). Once again, SMaSH outperformed the benchmarked linear methods.

**Table 2:** Marker gene misclassification rates in organs of origin in early foetal development

Data-set	scGeneFit	RankCorr	SMaSH (DNN)	SMaSH (RF)	SMaSH (BRF)	SMaSH (XGBoost)
HFO skin versus kidney versus liver	(13.9, 0.85)	(5.2, 0.95)	(1.1, 0.99)	(1.4, 0.99)	(1.8, 0.98)	(1.2, 0.99)

Performance in early foetal organ data, including the four different models implemented in SMaSH. All metrics are summarised as (*M*, $F_{1}$) tuples. The top 2 performing models are indicated in bold for each data-set

*HFO* Human foetal organs

## Discussion

The SMaSH framework is a new methodology for determining marker genes from large scRNA-seq data-sets, for both general and more specific user-defined cell annotations (e.g a few broad cell types vs. many cell sub-types). SMaSH has been designed as a specific software package for users involved in complementary scRNA-seq and STx biological analyses, using the data from the former to determine optimal genes for better performing analyses in the latter. Knowledge of annotations is essential to the running of the algorithm.

We find that SMaSH produces unique markers which better classify data-sets of a variety of sizes and complexities. SMaSH yields markers which, when used to reconstruct the original annotations in each data-set, possess consistently lower misclassification rates. This uniqueness applies to data-sets of varying granularity, as demonstrated by running SMaSH on separate human lung and mouse brain data-set in two modes. These are ‘broad’ cell classification of 7 different types for lung and 9 for mouse brain, and cell sub-types from each broad cluster leading to 34 distinct classifications of the lung cells and 31 distinct classifications for the mouse brain cells. Markers are ranked based on explainability parameters which capture the information gain which each gene adds to the supervised model. In particular, we observe that ranking marker genes based on Shapley values is effective for revealing the most explainable features in the neural network model, and note that this measure has yet to be explored in detail in applications of machine learning to problems in computational biology and transcriptomics. In addition to benchmarking SMaSH across several different single-cell sequencing data-sets, we also explicitly evaluated the performance of the mouse brain markers on corresponding mouse brain 10X Visium (see Additional file 1). We find that SMaSH is able to predict marker genes which capture the tissue behaviour of highly proliferating cells (such as astrocytes, present in both grey and white matter), and which also identify cell types which are known to be highly-localised to tissue compartments of the brain, such as identifying a highly localised compartment rich in endothelial cells close to the lateral ventricle around the hippocampus, and the precise reconstruction of a central tissue region rich in oligodendrocytes. Put together, we have therefore demonstrated that robust and interesting biological statements can be made using STx data in conjunction with the calculated SMaSH markers.

From the user perspective, we encourage broad investgation of the different models implemented in SMaSH. Based on the studies in this paper, we recommend using the feedforward neural network in the first instance, cross-checking the results with the XGBoost model, which for certain data-sets was observed to offer comparable or improved classification and marker gene identification.

## Conclusions

We have developed and presented SMaSH, a novel method for calculating marker genes in scRNA-seq data. We used non-linear methods as captured by two user-specified *network* and *ensemble* modes.SMaSH is available as a fully-integrated algorithm with ScanPy, making use of the AnnData object structure, common to many ‘big data’ analysis pipelines in single-cell computational biology. SMaSH has relevance for testing and confirming the presence of specific cell types in sequencing data, based on speculative annotations derived by the analyser, and also can be rolled out to assist in the design of downstream analyses and experiments. A notable application of this latter purpose is spatial transcriptomics, e.g. in situ sequencing, where 100–200 marker genes may be required for designing padlock probes. We summarise the SMaSH framework in a publicly-accessible webpage (see pypi), including self-contained notebooks where interested users can see example implementations for several data-sets mentioned in this paper (see GitLab repository). We recommend SMaSH as a standard component to a downstream analysis pipeline of scRNA-seq data where key genes must be extracted. Particularly users should bear applications to spatial transcriptomics or related techniques in mind. We hope that SMaSH serves as a welcome software simplification to the community, providing a natural extension to existing scRNA-seq bioinformatics pipelines.

## Methods

The SMaSH framework (Fig. 1) is divided into four stages, beginning from the user-defined input AnnData [9] object which contains the raw scRNA-seq counts in a matrix of dimensionality determined by the number of barcoded cells and unique genes in the data-set. The user must also provide a vector of target outputs to map each barcoded cell onto, with values corresponding to classes of the problem in question, e.g. a vector of annotations of each barcoded cell into a particular biological type. We stress that the choice of annotations is user-specific and highly-dependent on the problem of interest: SMaSH will work with any set of biologically-motivated annotations for gene calculation, although in this paper we will mainly focus on cell type annotations known *a priori* by the user, as this question is most appropriate for the downstream application of spatial analysis. SMaSH then extracts markers by analysing the counts and targets in a supervised machine learning classification task, where the most important markers map to the most important features for classifying cells according to the user’s required target annotations, and selects the most important genes for describing each class based on feature ranking with information-theoretic metrics.

### SMaSH algorithm

#### SMaSH filtering step 1: gene filter

The input cell-gene counts are first optionally batch-corrected using Harmony [23], and general genes connected to mitochondrial activity [24], ribosomal biogenesis [25], cell-surface protein regulation of the immune system, and biological housekeeping are removed. Genes which are lowly and highly expressed are further filtered out, so that only those which are expressed in greater than 30% of the classes of interest and in less than 75% of cells with more than 50% of the classes of interest are retained by default. This final filter guards against additional batch-specific effects, such as a particular gene not being expressed uniformly across most various different independent biological samples comprising the data-set of interest. The 30% and 75% thresholds are based on empirical checks to ensure that genes which are expressed in the classes of interest are retained, and may be modified by the user.

#### SMaSH filtering step 2: inverse PCA

The filtered matrix of cells and genes is then dimensionally-reduced using principal component analysis (PCA) [26] applied to each gene as a unique feature and each cell as an observation. The PCA is then inverted and the top 20 genes in each principal component explaining up to 80% of the overall variance in the data are retained. This additional feature guards against genes which would add very little extra information about the variance of expression profiles in the data and speeds up subsequent training of the model.

#### Main SMaSH model

The remaining genes are then ranked according to one of our machine learning classification modes: *ensemble* (RF, BRF, and XGBoost [12] as the default) and *network*. Details of the cross-validation on training, validation, and test sets are described in *Machine Learning Cross-validation*. Further details and additional benchmarking are provided in the Additional file (see Additional file 1: Fig. S1 and Table S3).

#### SMaSH post-modelling: ranking and heterogeneity

The final marker genes are calculated by ranking and sorting the genes according to their total Gini importance or mean Shapley value, where the mean Shapley value is used in the *network* mode. A set of relevant markers is produced for each class provided by the user from the initial vector of targets, where the top 5 markers per class are produced by default. A final heterogeneity check is conducted in the case that multiple samples are considered in the analysis, to make sure that the marker genes selected are also distributed uniformly in at least 70% of the set of samples considered in the data. For this latter check the user must ensure that sample information is provided as an observation in the original AnnData object.

### Benchmarking SMaSH

For each SMaSH model, linear model for benchmarking, and data-set, the top 30 markers are calculated for each annotation class then used as the only features in a *k*-nearest neighbours classifier for mapping each cell back to its original annotation. Support vector machine classifiers was used as an independent cross-check of the *k*-nearest neighbours performance, and these results are summarised in the Additional file. The misclassification rates, *M*, and associated confusion matrices for recovering the original ground-truth annotations were evaluated on each data-set and model. The average $F_{1}$ score was also calculated as the average harmonic mean of the precision and recall for each cell type classification, which is a more indicative performance metric for multi-classification problems than the more widely-known true-positive and false-negative rates. Note that we separately evaluated all metrics for each separate class, as well as computing the average value across all classes. These performance metrics may be formally defined as:

$$\begin{aligned} M = \Bigg \langle 1 - \frac{C_{i}}{P_{i}} \Bigg \rangle _{i \in {\mathcal {C}}} \end{aligned}$$

and

$$\begin{aligned} F_{1} = \Bigg \langle \frac{2}{\frac{1}{\mathcal {R}} + \frac{1}{\mathcal {P}}} \Bigg \rangle _{i \in {\mathcal {C}}}, \end{aligned}$$

where $C_{i}$ and $P_{i}$ denote the number of correct predictions and total predictions of class *i* from the *k*-nearest neighbours classifier respectively, $\mathcal {R}_{i}$ and $\mathcal {P}_{i}$ are the respective recall and precision of that classification, and the $\langle \rangle _{i \in {\mathcal {C}}}$ denotes averaging over all classes *i* belonging to the set of annotations $\mathcal {C}$ provided by the user. Lower misclassification rates (tending to 0) and higher average $F_{1}$ scores (tending to 1) indicate better performance of a given model.

### Machine learning cross-validation

SMaSH makes use of test, train, and validation sets. All machine learning studies described in this paper (the training and testing of the SMaSH models, and the *k*-nearest neighbours and support vector machine classification of the SMaSH markers) were validated in the following way: each data-set was divided in to a 80:20 train:test split. Training was only performed on the training data-set. The training data-set was cross-validated by constructing additional validation sets using the *k*-fold validation approach, where 5 folds were used (i.e. we independently evaluated performance metrics in a 80:20 training:validation split of the initial training data, permuted across the 5 batches with resampling). This *k*-fold cross-validation was done in a stratified manner, to ensure that fair representations of each class were selected in every training and validation set. The cross-validated loss functions and accuracies then allowed for overfitting to be checked against (see Additional file 1: Fig. S1 for an example of this in the training of the SMaSH DNN on mouse brain data). Additional checks for overfitting were performed for different models: applying dropout to the neural network, regularisation with kernel functions to the support vector machine, and early stopping in the neural network training. The support vector machine classifier acted as an additional cross-check of the *k*-nearest neighbours classifier when studying the optimal SMaSH genes to the genes calculated by other methods, and the results are reported in the Additional file 1: Table S3.

## Supplementary Information

**Additional file 1.** Supplementary Material for SMaSH: A scalable, general marker gene identification framework for single-cell RNA-sequencing.

## Data Availability

We considered several publicly-available data-sets in this study: the Zeisel [18] data covering a small population of mouse brain cells, a data-set based on CITE-seq technology [19], a mouse brain single-nucleus RNA-sequenced data-set [17], a healthy foetal liver data-set [21], and Paul15 stem cell data [20]. We also considered an extension of the foetal liver data-set covering skin and kidney cells which is again available at [21]. These data-sets were modified from their originals to include the cell type annotations in a single AnnData object, for use directly with the tutorials to accompany the release of SMaSH. These modified data-sets are available on a self-contained MEGA cloud here. The 10X Visium mouse brain data was obtained from the complementary single-nucleus RNA-sequenced data, where the Space Ranger toolkit was used to store the final Visium spots and genes expression profile and perform the genome alignment, and all spatial images of the brain were publicly available from the same result [17]. The final Visium data is available through relevant S3 buckets here. The runtime and memory allocation performance of SMaSH with these different data-sets was performed with the following CPU and RAM requirements—RAM: 32 GB 2133 MT/s DDR4; CPU: Intel(R) Xeon(R) CPU E7-8891 v3 at 2.80 GHz. The complete SMaSH implementation, including several full examples of how to use SMaSH and reproduce the results in the paper are available under the Cvejic group GitLab: https://gitlab.com/cvejic-group/smashhttps://gitlab.com/cvejic-group/smash. SMaSH is available under PyPI as the smashpy package (pip install smashpy), named to distinguish it from similarly-named existing packages.
