# Supplementary material for: SMaSH: a scalable, general marker gene identification framework for single-cell RNA-sequencing
Source: BMC Bioinformatics. 2022 Aug 8;23:328. doi: 10.1186/s12859-022-04860-2 (PMC9361618; doi:10.1186/s12859-022-04860-2)
Supplement: Supplementary file 1 — Additional file 1. Supplementary Material for SMaSH: A scalable, general marker gene identification framework for single-cell RNA-sequencing. [file 12859_2022_4860_MOESM1_ESM.pdf]

Supplementary Material for SMaSH: A scalable, general marker gene identification framework for single-cell RNA-sequencing

Michael E. Nelson[1,2,3,4,*,†], Simone G. Riva[2,3,4,5†], Ana Cvejic[2,3,5,*]

[1]European Bioinformatics Institute, Wellcome Genome Campus, Cambridge, CB10 1SD, UK

[2]University of Cambridge, Department of Haematology, Cambridge, CB2 0AW, UK

[3]Wellcome – Medical Research Council Cambridge Stem Cell Institute, Cambridge, CB2 0AW, UK

[4]Open Targets, Wellcome Genome Campus, Cambridge, CB10 1SA, UK

[5]Wellcome Sanger Institute, Wellcome Genome Campus, Cambridge, CB10 1RQ, UK

∗Corresponding authors: nelson@ebi.ac.uk, as889@cam.ac.uk

†Equal contributions

SMaSH Model Details and Cross-validation

The deep neural network is implemented with the `Keras` API, and its architecture determined by Bayesian hyperparameter optimisation with a Tree-structured Parzen estimator as implemented in the `Hyperas` framework where we started from a parsimonious set of different architectures. The network takes all filtered genes as unique input nodes, propagating weights through two hidden layers of 32 and 16 nodes respectively, each applying batch normalisation and sigmoid activation to the output. To aid regularisation, node drop-out is applied on a randomly selected 20 % and 10 % exiting the respective hidden layers. The user annotation classification is achieved by passing this output through a dense final layer of size equal to the number of unique annotations and a softmax function. Our decision to optimise over simple architectures is motivated by the observation that Shapley values are evaluated in exponential time, and therefore calculation of gene importance would slow down considerably using more complex architectures. The ensemble learners are implemented using the extensive `scikit-learn` library, and `imblearn` in the case of the BRF. Hyperparameter optimisation was performed using a greedy grid search across a large set of possible hyperparameters for each

learner, selecting the parameters with the largest F_1 score on the test-train split human lung cancer data-set, where a validation set from the initial testing set was selected to check for learning convergence and possible overtraining. The Random Forest model uses 100 estimators with no requirement on depth, and class weights are applied to the input data to reduce the impact of imbalanced samples. The Balanced Random Forest uses 200 estimators with a maximum depth of 50, and is also initialised with class weights and an 'all' sampling strategy. XGBoost uses 200 estimators with a maximum depth of 9, a softmax objective function (reducing to a logistic in the case of binary classification) with a 0.25 learning rate, and gradient boosting implemented through the Dart procedure. These ensemble learner parameters were obtained through a greedy grid search scan of the parameter space, training, testing, and validating each set of parameters on the lung cancer data-set to optimise the F_1 score.

As described in the main text, every model training instance is validated using 5-fold cross-validation: this applies to the training of SMaSH models to learn relevant markers, and the evaluation of the subset of those markers using k-nearest neighbours and support vector machine classifiers. Example cross-validation is illustrated for the SMaSH DNN model on the mouse brain data-set in Figure S1. At each fold, constructed from permuting the original training set with replacement in a stratified manner so as to sample all mouse brain cell classes, the loss function is evaluated over up to 100 epochs for the new training and validation sets, 80 % and 20 % of the original training data respectively. Early stopping after approximately 25 epochs is applied to prevent overfitting, together with regularisation of the DNN with dropout as applied to the new training set (this regularisation has the effect of increasing the training loss; dropout is not applied at the validation step, hence typically slightly smaller losses for that data are observed). At each fold, similar loss behaviour is observed in the training and validation sets, with a minimum for each obtained at the epoch of early stopping. The model accuracy of the training and validation sets, relative to the original test set, is also evaluated at each epoch per fold. In conjunction with the minimum loss, the accuracy also converges to close to 1in both sets at the epoch of early stopping, where the performance for fold 5 is shown in Figure S1**B)**.

SMaSH selects biologically meaningful and well-motivated marker genes

To investigate that SMaSH selects biologically versatile marker genes, we cross-checked the top markers per cell type and cell sub-type calculated for the mouse brain data across relevant literature. We have studied example markers calculated with SMaSH for the broad cell types, their cell type and function, and existing references in literature, confirming that SMaSH correctly learns biologically robust and interesting lists of marker genes relevant to the underlying neurobiology.

To investigate that SMaSH selects biologically versatile marker genes, we cross-checked the top markers per cell type and cell sub-type calculated for the mouse brain data across relevant literature. Table S1 summarises several example markers calculated with SMaSH for the broad cell types, their cell type and function, and existing references in literature, confirming that SMaSH correctly learns biologically robust and interesting lists of marker genes relevant to the underlying

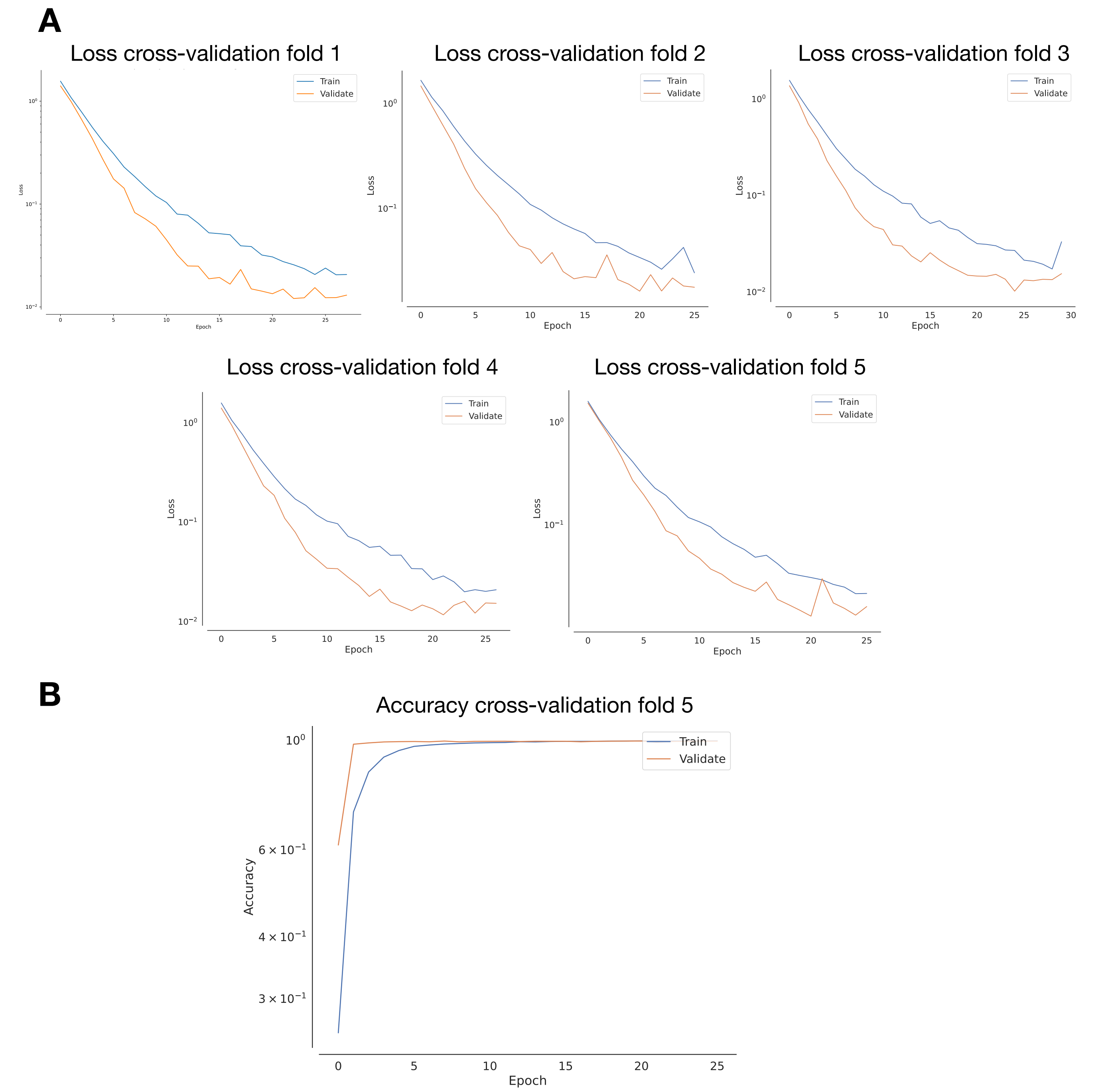

Figure S1. 5-fold cross-validation of the SMaSH DNN on mouse brain. **A)** Loss function (categorical cross-entropy); and **B)** the fold 5 accuracy as a function of the training and validation epochs, through to early stopping.

Cell type	Marker gene	Function
Astro	Slc1a2	Plays a role in neurodegeneration and synaptic plasticity and has been shown to associate with presenilin 1 in neurons and astrocytes, and has important implications in maintenance of glutamate homeostasis and amyloid $A\beta$ pathology.
Astro	Wdr17	Has enhanced expression in the human brain, parathyroid gland, and pituitary gland. Its function in the brain is not studied but its role in retinal disease is relatively more studied.
Endo	Bsg	Expression is detectable in vascular endothelial cells within single cell RNA sequencing data-sets derived from multiple tissues in healthy individuals.
Endo	Rundc3b	Enhanced cell type expression in endothelial cells is known, although its function in relation to endothelial cells is not studied.
Ext	Grin2b	Disruption impairs differentiation in human neurons.
Ext	Nrg3	Promotes excitatory synapse formation on hippocampal interneurons
Inh	Meg3	Long non-coding RNA MEG3 functions as a competing endogenous RNA to regulate ischemic neuronal death by targeting miR-21/PDCD4 signaling pathway.
Inh	Galntl6	This gene is a marker of neurons validated by ATAC-seq.
Micro	Inpp5d	Selectively expressed in brain microglia and has been reported to be associated with late-onset Alzheimer's disease.
Micro	Arhgap45	This gene was found to be a Alzheimer's disease-specific core human microglial signature gene.
Nb	Igfbpl1	Known to be specifically expressed in neuroblast stages, and various neuroblast populations
Nb	C130071C03Rik	Uncharacterised protein.
OPC	Tnr	Can potentially promote OPC adhesion and differentiation.
Oligo	Plp1	An oligodendrocyte myelin-rich tetraspan membrane protein and aberration of the Plp1 gene is known to be responsible for dysmyelinating Pelizaeus-Merzbacher disease.
Oligo	Prr5l	Its mRNA expression were found in myelinating oligodendrocytes in the brain.

Table S1. Marker gene functions for the broad mouse brain cells. Example markers genes across different broad cell types identified by SMaSH, together with known biological functions in the literature. Shortened cell type names correspond to astrocytes (Astro), microglia (Micro), endothelial cells (Endo), excitatory neurons (Ext), inhibitory neurons (Inh), neuroblasts (Nb), oligodendrocytes (Oligo), and oligodendrocyte precursors (OPC).

neurobiology. This list is far from exhaustive but the scope of the marker gene functions demonstrates that markers with a variety of biological functionality can be selected from a rich scRNA-seq data-set like the mouse brain.

The markers calculated for the classification of foetal organs, as reported in the main text, were also cross-checked in the same way using available literature, and were found to possess biologically relevant functions for their organ classification. These biological interpretations are summarised in Table S2.

Benchmarking SMaSH using support vector machines to cross-check k-nearest neighbours

We cross-checked the performance of SMaSH using the top 30 markers per cell type in the k-nearest neighbours classification exercise, with ground truth provided by the user's original annotation vector, by repeating the exercise on different

Organ	Marker gene	Function
Kidney	RBP1	Retinol binding protein (RBP) is a low molecular weight protein belonging to the lipocalin super family and mainly synthesized in the liver. Its main function is to transport retinol (vitamin A).
Kidney	ID3	A functional ID3 influences susceptibility to kidney disease and prevents glomerular injury by regulating local chemokine production and inflammatory cell recruitment.
Kidney	GPC3	Plays a role in cell growth and differentiation. Mutations of the GPC3 gene are responsible for Simpson-Golabi-Behmel syndrome, which is characterized by anomalies of postnatal overgrowth and an increased risk of developing pediatric malignancies, mostly Wilms tumor. GPC3 is expressed in the fetal uteric bud and collecting system in a time-specific manner. Human fetal tissue corroborates a developmental function in the kidney as renal tissue from patients with congenital renal dysplasia has decreased expression of GPC3.
Kidney	CRABP2	CRABPs are low-molecular-weight, intracellular proteins that act on RA-induced transcriptional activity, maintaining an adequate RA metabolism (RA - retinoic acid). CRABP2 transports RA from the cytoplasm to the nucleus, promoting RAR ligation and RXR heterodimer formation. Upregulation of CRABP2 has been reported in the blastema of nephroblastomas during the investigation of genes related to nephrogenesis.
Kidney	WFDC2	The WFDC2 gene encodes for a putative serine protease inhibitor that is upregulated in human and mouse fibrotic kidneys and is elevated in the serum of patients with kidney fibrosis.
Liver	HBA2	Involved in oxygen transport from the lung to the various peripheral tissues. Deletion leads to alpha thalassemias.
Liver	HBA1	Involved in oxygen transport from the lung to the various peripheral tissues. Deletion leads to alpha thalassemias.
Liver	ALB	Human serum albumin is synthesized exclusively by hepatocytes. Albumin is responsible for about 70 % of plasma oncotic pressure. Human serum albumin may play an important role in modulating innate immune responses to systemic inflammation and sepsis.
Liver	APOA2	APOA2, the second major HDL apolipoprotein. Moreover, studies in either human or murine apoA-II transgenic mice. and apoA-II knockout mice, indicate that apoA-II is involved in plasma clearance of triglyceride-rich lipoproteins; influences plasma levels of free fatty acids, glucose, and insulin; and affects adipose mass, which suggests a role of apoA-II in insulin sensitivity and fat homeostasis.
Liver	AC104389.1	Long non-coding RNAs (lncRNAs) are emerging as critical biological mediators in the normal functioning of the liver. Aberrant expression of lncRNAs is associated with metabolic diseases, fibrosis, and malignancies involving the liver.
Skin	COL1A1	Type I collagen is the major protein in bone, skin, tendon, ligament, sclera and cornea tissues, blood vessels, and hollow organs.
Skin	COL1A2	Type II collagen is found in articular cartilage.
Skin	COL3A1	Type III collagen is often associated with Type I collagen and is a major protein in skin, vessels, intestine, and the uterus.
Skin	DCN	Decorin is a multifunctional proteoglycan involved in several biological processes, like matrix organization. Decorin deficient matrix displays altered sulfate levels that affect growth factors involved in wound healing.
Skin	LUM	A keratan sulfate small leucine-rich proteoglycan (SLRP) localized to the ECM, and known to regulate collagen fibrillogenesis in connective tissues, e.g. cornea, tendon and skin. LUM binds fibrillar collagens, and regulates collagen fibril thickness and interfibrillar spacing, important for tissue integrity and corneal transparency.

Table S2. Marker gene functions for the classification of foetal organs. Example markers genes across different foetal organs (skin, liver, kidney) identified by SMaSH, together with known biological functions in the literature.

Data-set	scGeneFit	Wilcoxon	RankCorr	SMaSH (DNN)	SMaSH (RF)	SMaSH (BRF)	SMaSH (XGBoost)
Human lung cancer (broad)	(24.3, 0.75)	(14.7, 0.86)	(10.2, 0.90)	(5.7, 0.94)	(6.4, 0.94)	(7.8, 0.92)	(6.4, 0.94)
Mouse brain (broad)	(36.9,0.62)	(10.6, 0.90)	(10.5, 0.90)	(0.4, 1.0)	(1.0, 0.99)	(1.5, 0.99)	(0.5, 0.99)
Zeisel	(8.5, 0.91)	(12.5, 0.87)	(5.4, 0.95)	(4.0, 0.96)	(3.4, 0.97)	(3.4, 0.97)	(3.7, 0.96)
CITE-seq	(15.4, 0.86)	(18.5, 0.81)	(16.9, 0.86)	(6.0, 0.94)	(6.7, 0.94)	(7.1, 0.93)	(7.7, 0.93)
Paul15	(28.1, 0.73)	(32.7, 0.66)	(23.9, 0.77)	(16.8, 0.83)	(18.1, 0.82)	(23.8, 0.77)	(18.4, 0.82)
Human foetal liver	(61.7, 0.36)	(40.0, 0.59)	(6.4, 0.94)	(5.7, 0.95)	(5.7, 0.95)	(5.8, 0.95)	(6.3, 0.94)

Table S3. Marker gene misclassification rates in broad cell types with Support Vector Machine classifier. The average misclassification rates, M, in percent, and the weighted average F_1 scores across all classes (cell types) for each data-set and framework, including the four different models implemented in SMaSH. All metrics are summarised as $(M,\ F_1)$ tuples. All SMaSH models outperform existing approaches across all data-sets.

classifiers. We considered the support vector machine classifier, implemented with default settings from scikit-learn. The performance is summarised in Table S3 respectively. The improved performance of SMaSH over scGeneFit and RankCorr is noted, providing further justification for the k-nearest neighbour results we quote in the main text.

Sensitivity of SMaSH performance to different numbers of top marker genes in the final classification

The performance of SMaSH was first evaluated using the top 30 marker genes per cell type, across different data-sets. As an additional check, we evaluated the performance of SMaSH as a function of different numbers of top marker genes selected for each cell type. We considered the human lung cancer data-set, evaluating the marker genes learned from the SMaSH DNN model for classifying each cell type based on the accuracy and F_1 score for recovering the original annotations. We studied the classification performance from selecting only the top 5 markers per lung broad cell type, to the top 150 per lung broad cell type (Figure S2), and observe variations in accuracy and F_1 at the few percent level at most. We therefore conclude that the addition of many extra top markers does not improve the classification quality, and in fact degrades the final performance due to the addition of redundant information in the classification task. Moreover, this study provides further justification to our decision of classify based on the top 30 markers per annotation class in the benchmarking of the SMaSH models, where we here observe that 30-40 markers per cell type corresponds to the highest accuracy (therefore lowest misclassifciation rate) and F_1 score. This optimal performance indicates that SMaSH, from the point of view of applications in spatial transcriptomics, can reliably propose 30 top markers per cell type, easily giving rise to 50-400 marker gene across the entire data-set, depending on how many cell types are being considered in the final study. For comparison, running classifications of scGeneFit and RankCorr with more marker genes does not significantly improve their performance to be competitive with SMaSH. For scGeneFit we re-calculated its human lung cancer misclassification using 100 top markers and found an improved result of 9.7 % (F_1 of 0.90) which still significantly under-performs the SMaSH classification which runs with a significantly smaller number of markers. The corresponding

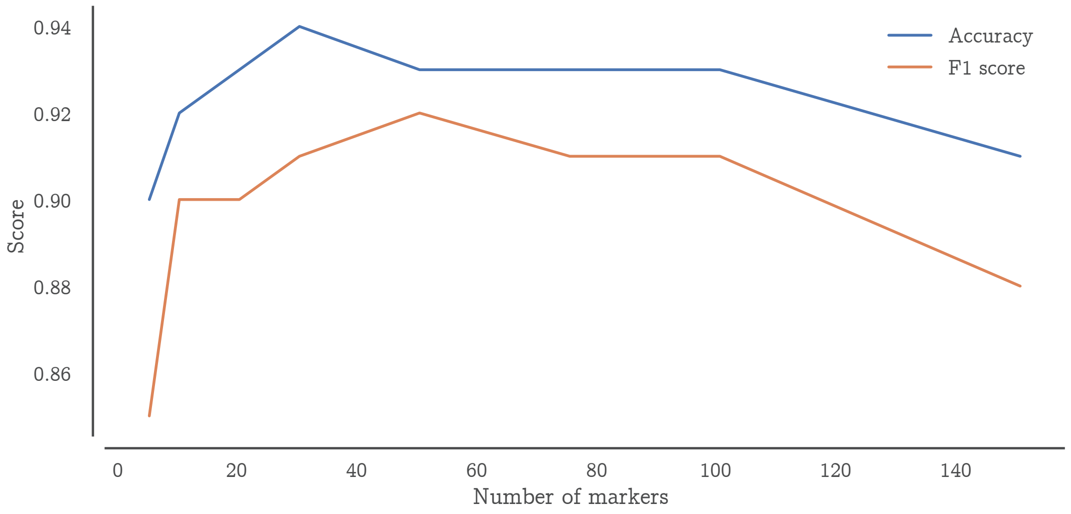

Figure S2. SMaSH lung cell type classification performance with varying numbers of top marker genes per cell type. The accuracy (1 − misclassification rate) and F_1 sensitivity to the number of top SMaSH markers selected per cell type in the human lung cancer classification task for recovering the original user broad cell types are shown.

check with RankCorr generated a misclassification of 10.6 % (F_1 of 0.89), very similar to the original result[1].

SMaSH for measuring over- and under-clustering

An additional application of SMaSH is for the detection of over- and under-clustered RNA-seq data. An optimal clustering is necessary in order to isolate distinct, biological cell phenotypes in a sample. Such phenotypes should have a number of distinct marker genes i.e. marker genes which are not strongly shared between other clusters in the data. We can therefore use this *sharing factor* of a particular marker gene to estimate whether a given configuration of clusters is over- and under-clustered, and hone in on the biological optimum configuration. We define the sharing factor (SF) of marker gene g_i in cluster c_j to be:

$$\mathrm{SF}_{ij} = \frac{E_{g_i}^{c_j}}{\sum_k E_{g_i}^{c_k}}, \tag{1}$$

where $E_{g_i}^{c_j}$ is the log-scaled and normalised expression of g_i in c_j, and the k clusters in the denominator is the set of all clusters in the data, including j.

For the over-clustered case, the sharing factor would be close to zero across nearly all clusters; the under-clustered case would have a maximal sharing factor close to 1 between all clusters. Based on this, the more optimal clusterings would be expected to have a reasonably large sharing factor, whilst still not being pathologically under-clustered (which would generate higher sharing factors across all markers and clusters). By evaluating this technique on the mouse brain

[1]Because of the design of the RankCorr algorithm, we can not an arbitrary select the number of top ranked markers to use in the classifier, so we classified based on the maximum number of top markers it could obtain, which is 32.

snRNA-seq, we estimate sharing factors of 0.7-0.8 to correspond to reasonably optimal clusterings. To demonstrate this, we reclustered the mouse brain snRNA-seq: the raw counts were normalised and scaled, then a neighbourhood graph was built before finally applying Leiden clustering at different resolutions to generate different sets of clusters, ranging from the pathologically small (2 clusters, clearly under-clustering) to the pathologically large (more than 70 clusters, clearly over-clustering). These results are displayed in Figure S3. As expected the over-clustered cases (**A, B, C, D**) have smaller factors across nearly all clusters, and the under-clustered case (**F**) has factors close to 1. The more optimal clustering (**E**) represents a more reasonable compromise in the sharing of marker genes (note that the final published annotations defined 9 broad cell type clusters in the brain, closest to case E in our example). Whilst this study is not conclusive, it represents a possible way of extending the usual SMaSH utility to assess cluster quality based on the key marker genes resulting from a particular clustering configuration. This is not the main purpose of SMaSH but it does present an interesting bonus application for future RNA-seq studies.

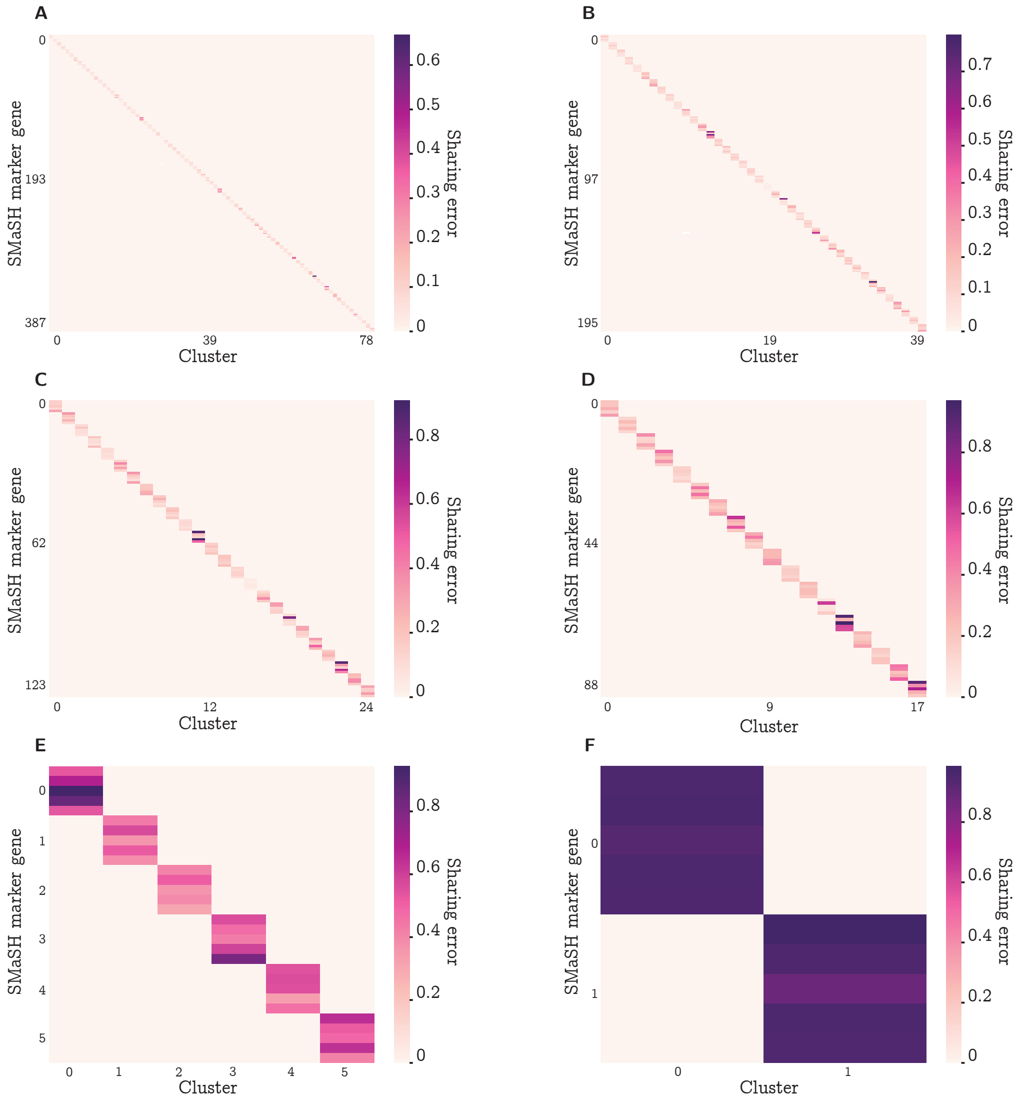

Figure S3. SMaSH as a method for measuring clustering performance. Example sharing factors for different clustering scenarios in the mouse brain snRNA-seq.

SMaSH markers identify highly-specific tissue compartments in the mouse brain STx data

In addition to studying the gene expression of SMaSH markers across different cell types and data-sets, we evaluated the performance of marker genes calculated by SMaSH from publicly available scRNA-Seq data set on corresponding 10X Visium sections. The 10X Visium protocol was applied to fresh-frozen mouse brain tissue, generating a set of whole transcriptome spots where each spot corresponds to a capture area of up to 10 cells. We focus on three cell types: astrocytes, oligodendrocytes, and endothelial cells. Astrocytes are found in both white and grey matter, and therefore would be present across much of the brain tissue, but can also be sparsely located in specific tissue compartments. This is contrasted with oligodendrocytes, where recent studies have demonstrated that they are localised to the central white matter region, and endothelial cells, which localise to lateral ventricles e.g. near the hippocampus. Figure S4 shows the expression profile of one of the top three ranking SMaSH markers for the astrocyte (Slc1a2), oligodendrocyte (Plp1), and endothelial cells (Bsg) in the mouse brain 10X Visium sections. Comparing with the corresponding anatomical diagram (Figure 5A) the astrocyte marker Slc1a2 (Figure S4**B**) shows an expression pattern typical of the astrocytes. Furthermore, the Plp1 marker (Figure S4**C**) expression profile reconstructs the white matter region in the centre of the brain, as well as the upper right flanking region about the hippocampus, where oligodendrocytes are localised. As expected there is essentially zero expression outside of this white matter region where oligodendrocyte density is significantly lower. Finally, the expression of endothelial marker Bsg overlaps strongly with the very small region corresponding to the lateral ventricle near the hippocampus (Figure S4**D**), where endothelial cells will strongly localise. The high expression profile at the few dots around within and around the ventricle are consistent with the high endothelial density exclusively at that region. Taken together, these results demonstrate that SMaSH is able to learn highly tissue- and cell type-specific markers for applications in the spatial measurement of cell populations.

SMaSH shows competitive performance in runtime and memory usage for large data-sets

In Figure S5 we show the runtime and memory performance of the feedforward neural network in SMaSH against the different benchmarked models and the standard Wilcoxon test as implemented in ScanPy. While SMaSH offers little performance improvements for runtime of code and memory allocation in small data-sets (Paul15), it can be seen that it modestly outperforms current approaches when analysing larger data-sets (mouse brain), motivating SMaSH as a scalable algorithm for more complex gene expression profiles ever more common in computational biology.

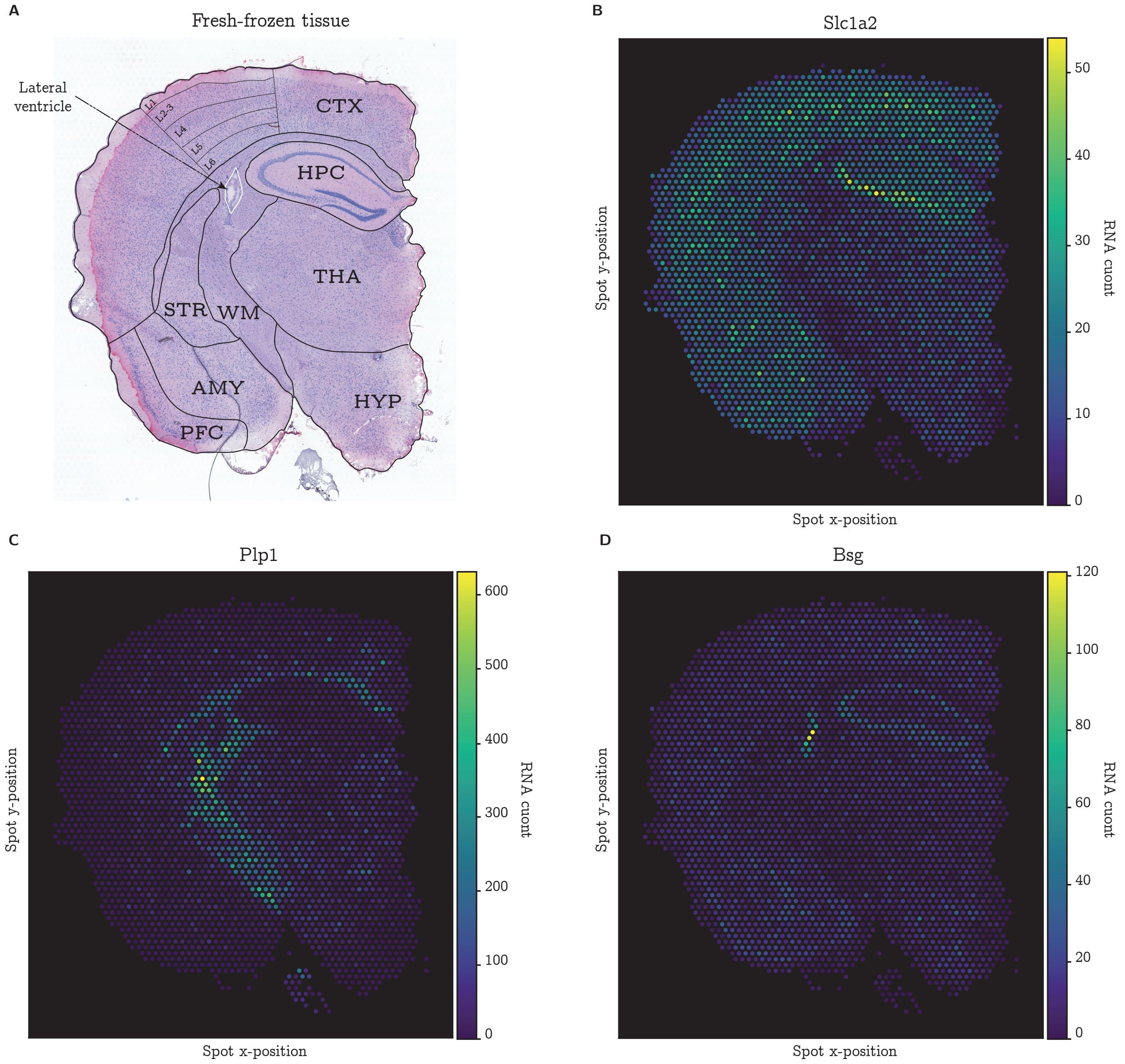

Figure S4. Mouse brain marker genes identify specific broad cell types in 10X Visium. Cross-section of mouse brain, with astrocyte, oligodendrocyte, and endothelial cell marker gene expression shown in the different colour scales (pre-mRNA count). **A)** The ground-truth anatomy of the brain, with white matter region (WM), lateral ventricle, hippocampus (HPC), thalamus (THA) hypothalamus (HYP), and other tissue compartments indicated. **B)** Slc12a, **C)** Plp1, and **D)** Bsg top markers for spatial expression profiles (for the astrocytes, oligodendrocytes, and endothelial cells respectively).

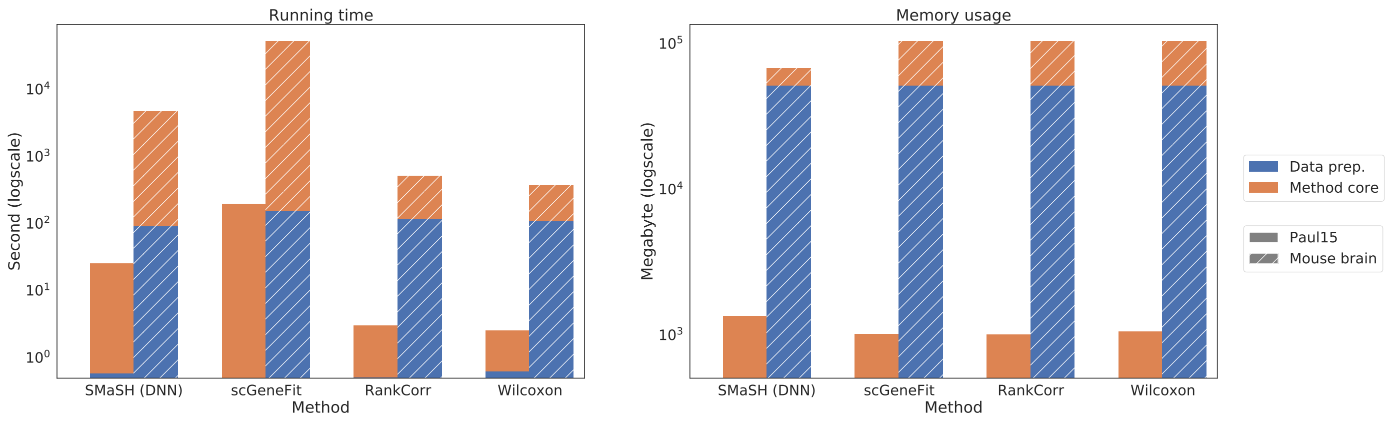

Figure S5. Runtime and memory performance The running time and memory usage time for running the neural network model in SMaSH, benchmarked against existing approaches.
